# Mesmerism

**Published:** 1890-06

**Authors:** 


### MESMERISM.

The well attested preternatural phenomena of somnambulism and animal magnetism so-called, are a stumbling block to the science which claims arbitrament over all things in the universe. Nothing is better evidenced than the mysterious occasional fact of direct action of mind upon mind, and of mind upon body (its own or another's, or even inanimate substance) without the intervention of any sensible medium of force. The proofs have multiplied until they have forced themselves on the attention of "science," and the French doctors have taken them up (*Sanitary Era*, 1888), and tried to fit them to a clumsy and inconsequent theory which they call "Hypnotism." Every body knows that a thing is made perfectly clear when a Greek name is put on it. The mysterious trance condition is explained by the resident surgeon of St. Barnabas Hospital, Minneapolis, Minn. He reports an operation of a very painful kind performed on a boy of seventeen years without the use of other anæsthetic than the power of "hypnotism." The patient was led to and from the operating table, and even assisted the operator by assuming any position ordered, yet suffered no pain, lay perfectly quiet two hours and then called for food, as the operator had suggested that he should do at a certain time.—*San. Era.*

